# Modified Liquid-Based Cytology Technique for Immunocytochemistry in Effusion Specimen

**DOI:** 10.31557/APJCP.2019.20.9.2611

**Published:** 2019

**Authors:** Natcha Patarapadungkit, Porntip Jangsiriwitayakorn, Surachat Chaiwiriyakul, Phannatorn Sirivech, Ratchaneekorn Thongbor, Em-Orn Phanomsri, Luxkana Nititarakul

**Affiliations:** *Department of Pathology, Faculty of Medicine, Khon Kaen University, Khon Kaen, Thailand. *

**Keywords:** Modified liquid-based cytology, immunocytochemistry, cytology, effusion

## Abstract

**Objective:**

Immunocytochemistry (ICC) of serous effusion is an important tool for the diagnosis of benign and malignant cells. Our aim was to develop a modified liquid-based cytological technique for ICC (i.e., a modified LBC).

**Methods:**

Serous effusions of 110 cases were collected for cytological examination: 50 were negative for malignancy albeit benign mesothelium was found, and 60 were confirmed metastatic adenocarcinoma according to the modified LBC preparation. The latter were stained for *EMA*, *Ber-EP4*, *Calretinin*, and *p63* then interpreted by both a cytotechnologist and a pathologist. A comparative analysis of the diagnostic results was conducted.

**Results:**

The results of the metastatic adenocarcinoma were 100% (60/60) positive for *EMA* and 91.7% (55/60) positive for *Ber-Ep4* but negative for calretinin and *p63*. Cases negative for malignancy were 100% (50/50) positive for calretinin but negative for carcinoma markers. The difference between ‘positive for metastatic adenocarcinoma’ and ‘negative for malignancy’ in ICC was statistically significant (p < 0.001).

**Conclusion:**

The current study demonstrated that a panel marker, comprising *EMA*, *Ber-EP4*, and calretinin can be used for differentiating between cases of metastatic adenocarcinoma and benign mesothelium. The serous effusion specimen collected by the modified LBC technique is an effective preparation method for ICC.

## Introduction

Malignant disease is one of the most common causes of pleural, pericardial, and peritoneal effusion. Malignant effusions usually represent disseminated malignancy and indicate an advanced stage of cancer. Many types of malignancy are caused by errant metastatic cells from nearby organs (lung, liver, stomach, or ovary) occupying serous cavities. Most metastatic malignancies are adenocarcinoma, while scant squamous cell carcinoma, small cell carcinoma, and other types of malignant cells are also found. Mesothelioma is a cancer originating from mesothelial cells lining the body cavity. Mesothelioma can be found in an effusion albeit rarely (Pereira et al., 2006). Malignant effusion indicating end-stage disease usually precludes surgical therapy of the primary malignancy, which has a significant impact on the follow-on clinical treatment decisions (Kim et al., 2010). Once a diagnosis of malignant effusion is established, the patient will likely receive chemotherapy or radiotherapy. Accuracy of the cytologic diagnosis of malignant effusion is thus extremely important (Shidham, 2007). The sensitivity and specificity of a cytomorphological diagnosis ranges between 52-84% and 89-92% (Motherby et al., 1999; Sen et al., 2015). 

A limitation of an effusion cytological diagnosis is being able to differentiate free malignant cells especially metastatic adenocarcinoma from reactive mesothelial cells because of their overlapping cytomorphologic features. The cytomorphologic features used to identify malignant cells include: nuclear pleomorphism, prominent nucleoli, irregular nuclear membrane, cytoplasmic vacuolization, large cellular aggregates, papillary fragments, cell balls, and cell-in-cell engulfment. These features, however, have limited use in effusion because they may also be present in reactive mesothelial cells. In many cases, a cytological diagnosis cannot be concluded on the basis of morphology alone, so diagnostic accuracy must be enhanced through ancillary techniques (Lyons et al., 2008). 

Immunocytochemistry (ICC) is an ancillary technique that uses an antibody that binds to a unique sequence of amino acids of the target protein; so as to identify proteins and molecules in cells and tissues that are then viewed under microscope. The technique is simple, cost-effective, and can differentiate mesothelial cells from malignant cells. Although less accurate grading and scoring may be a limitation of the method, the ICC technique is used for routine diagnosis of cancer in many laboratories (Metzgeroth et al., 2011). 

There are several cell preparation methods for ICC including direct smear, formalin-fixed paraffin-embedded (FFPE) cell block, and cytospin smear (Skoog and Tani, 2011). FFPE-cell block is widely used for diagnosis of serous effusion, but there is little information about its practical use. The modified liquid-based cytology (modified LBC) could be used on serous effusion for cancer diagnosis, as it uses ethanol and normal saline in its preparation Modified LBC is much less expensive than current commercial fixative solutions for liquid-based fixations, and it maintains cell preservation with a clean smear background (Jangsiriwitayakorn et al., 2018). The current study aimed to assess relatively inexpensive chemical liquid fixative preparation methods, and whether they can be used with an ICC technique to improve the quality of cytological diagnosis.

## Materials and Methods

Our study protocol was reviewed and approved by the Ethics Committee at Khon Kaen University, Thailand. Serous effusions of 110 cases were collected for cytological examination between August, 2014 and March, 2016 from patients at Srinagarind Hospital. Fifty cases were negative for malignancy and positive for benign mesothelium. Sixty cases were metastatic adenocarcinoma according to the modified LBC preparation. Effusions that met the criteria for sample effusion were sent for assessment (i.e., fresh native, sufficient number of neoplastic cells, and a minimum 3-5 mesothelial cells/HP). The effusion specimens were prepared using the modified LBC preparation after which ICC was performed. The immunomarkers for ICC staining used in the current study included *BerEp4*, *EMA*, *p63*, and *Calretinin*. The results of the ICC markers were determined EMA for carcinoma, BerEP4 for adenocarcinoma, Calretinin for mesothelial cell, and p63 for squamous cell carcinoma. 

*Processing of modified LBC preparation*

Effusion samples (10 ml/sample) were centrifuged at 1,600 rpm for 10 min. The supernatant was discarded and the protein washed with 0.9% normal saline solution. This was then centrifuged at 1,600 rpm for 5 min after which 50% ethanol was added at a 1:1 ratio. The specimen was incubated for 15 min at room temperature to induce blood hemolysis. The specimen was centrifuged for 5 min at 1,600 rpm, and re-suspended with 70% ethanol (the fixative solution).

*Processing of ICC*

The preserved 70% ethanol specimens were centrifuged for 10 min at 1,500 rpm at room temperature. Approximately 50 µl of the sediment was aspirated and transferred onto positively-charged slides. The slides were air-dried at room temperature after fixing (for at least 15 min) in 95% alcohol. The sample was dried on a horizontal surface for 60 min at room temperature before performing *EMA*, *BerEP4*, *Calretinin*, and* p63*. 

The positive control for the *EMA* and *BerEP4* were adenocacinoma cells confirmed by biopsy. The negative controls for both *EMA* and *BerEP4* were mesothelial cells (confirmed by biopsy) from a patient without any history of malignancy. The positive control for Calretinin were mesothelial cells (confirmed by biopsy) from a patient without any history of malignancy. The negative control for Calretinin were adenocacinoma cells confirmed by biopsy. The positive control for *p63* were basal cells from a cervical smear from a liquid-based cytologically-preserved specimen. The negative control for *p63* were mesothelial cells from a patient without any history of malignancy. The ICC reaction studies were performed using an automated Ventana BenchMark ULTRA immunostainer, using an UltraView Universal DAB Detection Kit (Roche; USA) as per the manufacturer’s instructions. Results of the immunocytochemical expression were used to determine the proportion of epithelial cells stained brown as follows: negative = no staining, < 10%, 10-50%, > 50% of positive cells (Ikedo et al., 2011). The immunocytochemical staining was considered a positive or a negative by the cytopathologist and pathologist.

## Results

*Patient data*

The samples were obtained from 110 patients, collected as serous effusions. The age of patients ranged between 27 and 89 years (mean 55.9). Overall, specimens included 54 pleural (49.1%), 50 peritoneal (45.5%), and 6 pericardial fluids (5.4%). The most common cause of benign pleural effusions in males was tuberculosis (4 cases; 26.7%) while in females it was pneumonia (3 cases; 20.0%). The most common cause of benign peritoneal effusions in males vs. females was cirrhosis (17 cases; 58.6% vs. 4 cases; 13.7%, respectively). The most common cause of benign pericardial effusion in males was chronic pericarditis (2 cases) vs. congestive heart failure in females (2 cases). Of the 60 malignant effusions, 39 were pleural effusions (65.0%) vs. 21 peritoneal effusions (35.0%). The most common cause of malignant pleural effusions in males was lung cancer (15 cases; 38.5%) vs. breast cancer in females (13 cases; 33.3%). The most cause of malignant peritoneal effusions in males was cholangiocarcinoma (4 cases; 19.0%) vs. ovarian cancer in females (8 cases; 38.1%).

**Figure 1:**
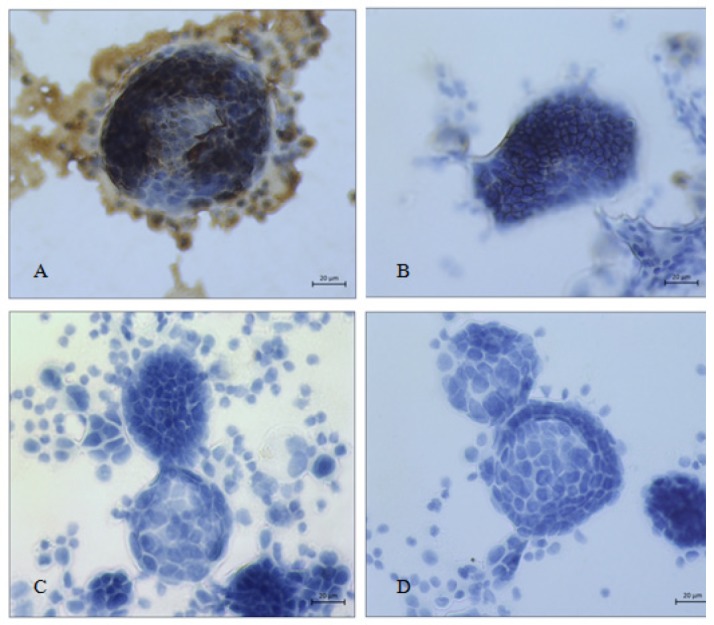
Metastatic Adenocarcinoma Cells of Breast in Pleural Effusion; A, B, *EMA* and *Ber-EP4* positive in malignant cells (x 400); C, D, Calretinin and *p63* negative in malignant cells (x 400)

**Figure 2:**
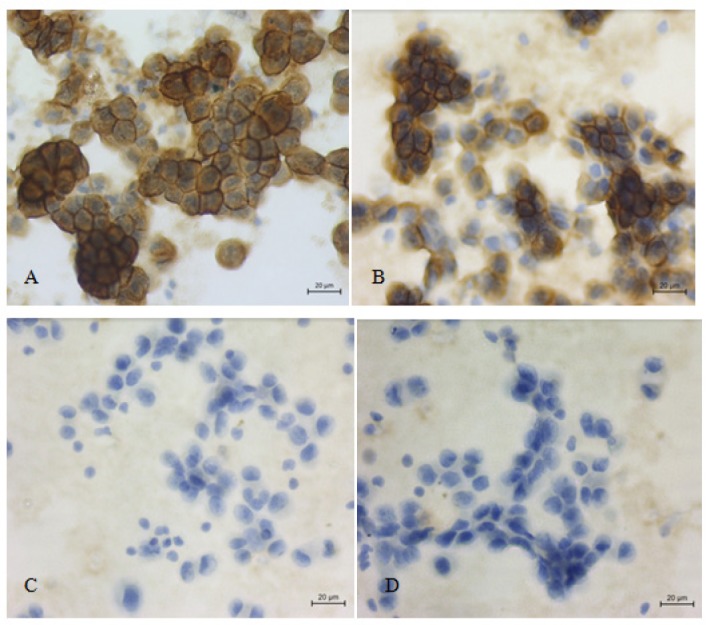
Metastatic Adenocarcinoma Cells of Lung in Pleural Effusion; A, B, EMA and Ber-EP4 positive in malignant cells (x 400); C, D, Calretinin and p63 negative in malignant cells (x 400)

**Figure 3:**
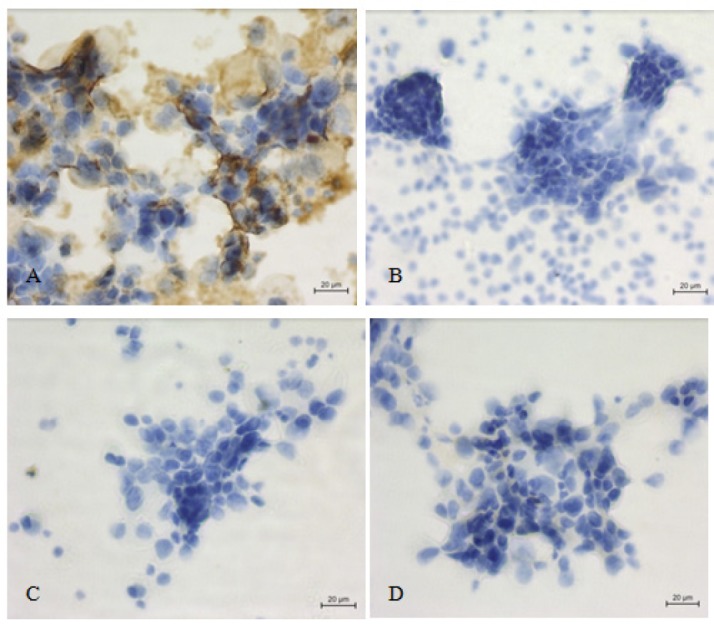
Metastatic Adenocarcinoma Cells of Breast in Pleural Effusion; A, *EMA* positive in malignant cells (x 400); B, C, D, *Ber-EP4*, Calretinin and *p63* negative in malignant cells (x 400)

**Figure 4:**
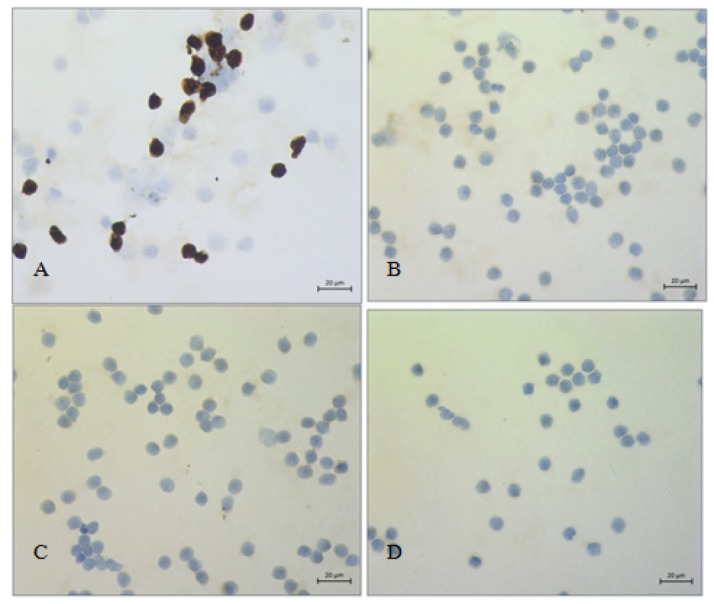
Reactive Mesothelial Cells of Cirrhosis in Peritoneal Effusion; A, Calretinin positive in reactive mesothelial cells (x 400); B, C, D, EMA, Ber-EP4 and p63 negative in reactive mesothelial cells (x 400)

**Table 1:** Expression Results for EMA, Ber-EP4, Calretinin, and p63 Staining

Diagnosis with Pap stain	Expression	Immunomarker			
		EMA	Ber-EP4	Calretinin	P63
Adenocarcinoma (60 cases)	-	0%	3 (5.0%)	60 (100.0%)	60 (100.0%)
	+/-	0%	2 (3.3%)	0%	0%
	1+	11 (18.3%)	17 (28.4%)	0%	0%
	2+	49 (81.7%)	38 (63.3%)	0%	0%
	Total positive	60 (100.0%)	55 (91.7%)	0 (0%)	0 (0%)
Mesothelial cells (50 cases)	-	50 (100.0%)	50 (100.0%)	0%	50 (100.0%)
	+/-	0%	0%	0%	0%
	1+	0%	0%	20 (40.0%)	0%
	2+	0%	0%	30 (60.0%)	0%
	Total positive	0 (0%)	0 (0%)	50 (100.0%)	0 (0%)

**Table 2:** Summary of Samples Positive for EMA, Ber-EP4, Calretinin, and p63

Diagnosis with Pap stain	Total	Positive (%) in immunocytochemistry antibody			
		EMA	Ber-EP4	Calretinin	P63
Adenocarcinoma	60	60 (100.0%)	55 (91.7%)	0%	0%
Mesothelial cells	50	0%	0%	50 (100.0%)	0%
	*P-values*	*P < 0.001*	*p < 0.001*	*P < 0.001*	*p > 0.999*

*Results for EMA, BerEP4, Calretinin, and p63 staining*

All 110 of the effusion samples (60 metastatic adenocarcinomas and 50 benign mesothelials) were prepared for ICC using the modified liquid-based preparation for *EMA*, *Ber-EP4*, *Calretinin*, and *p63*. 

The respetive immunocytochemical expression was used to determine the proportion of positive cell as follows: negative = no staining, +/- < 10%, 1+ = 10-50%, and 2+ > 50% of positive cells. 

The respective expression of *EMA* as a carcinoma marker at 1+ and 2+ was 11/60 (18.3%) and 49/60 (81.7%) for metastatic adenocarcinoma while there was no expression of any of the 50 benign mesothelial cases. Ber-EP4 is an adenocarcinoma marker for which the respective expression of *Ber-EP4* was no staining, ± < 10%, 1+ and 2+ in 3/60 (5.0%), 2/60 (3.3%), 17/60 (28.3%) and 38/60 (63.3%) cases of adenocarcinoma. *Ber-EP4* had no expression in any of the 50 cases of benign mesothelial. Calretinin is a mesothelial marker for which the respective expression in the 50 benign cases was 1+ and 2+ in 20/50 (40.0%) and 30/50 (60.0%) cases. Calretinin had no expression in the 60 cases of metastatic adenocarcinoma. *P63* is a squamous cell carcinoma marker and there was no expression of it among either the metastatic adenocarcinoma or the benign mesothelial cases (Table 1). 

We considered a result was positive if there were > 10% (1+ and 2+) of brown in the stained epithelial cells and negative if there was no staining or < 10% (±) of brown in the stained epithelial cells (Table 2). All 60 malignant effusions were classified as metastatic adenocarcinoma by morphology and most (55/60; 91.7%) were positive for *EMA *and *Ber-Ep4* while calretinin and* p63* were negative (Figures 1 and 2). A minority (5/60; 8.3%) of cases were positive for *EMA* while *Ber-Ep4*, *calretinin*, and* p63* were negative (Figure 3). 

A total of 50 benign mesothlial cases were classified based on morphology, and all were positive for *calretinin *while *EMA*, *Ber-Ep4*, and *p63* were negative (Figure 4). 

*EMA *is a carcinoma marker, and it was positive in all 60 cases of metastatic adenocarcinoma and negative in the 50 cases of benign mesothelial cells. The *Ber-EP4 *markers were mostly positive among the metastatic adenocarcinoma samples (55/60; 91.7%) and negative among the 50 benign cases. Calretinin is a mesothelial marker and it was positive in all 50 of the benign cases (100%). *p63* a squamous cell carcinoma marker and it was negative in all cases of metastatic adenocarcinoma (n=60) as well as the benign cases (n=50). The expression of* EMA*, *Ber-EP4*, and calretinin showed significant differences between adenocarcinoma and the benign mesothelial cells (p < 0.001) but the expression of p63 showed no significant difference between adenocarcinoma and the benign mesothelial cells (p > 0.999) (Table 2). 

## Discussion

The 110 effusions comprised 60 malignant and 50 benign effusions among which pleural effusions were the most common (39 or 65.0%), followed by peritoneal effusions (21 or 35.0%). Lung cancer was the most common disease among males (25.0%) followed by cholangiocarcinoma (6.7%); while breast cancer was the most common among females (21.7%) followed by lung cancer (15.0%). The remaining 5 cases (8.3%) of malignant effusions were from unknown diseases. Lung and ovary cancers were the most common malignancy in pleural and peritoneal effusions. Sen et al., (2015) similarly reported that lung cancer was the most common disease among males (21.6%) followed by cholangiocarcinoma (2.7%) while ovarian cancer was the most common disease among females (31.0%) followed by lung cancer (17.5%). In our study, among the 50 samples of benign mesothelium effusion, most were peritoneal effusion (58.0%) followed by pleural effusion (30.0%) and pericardial effusion (12.0%). Tuberculosis and cirrhosis were the most common disease in pleural effusion and peritoneal effusion, respectively. Congestive heart failure and chronic pericarditis were the most common diseases associated with pericardial effusion. Sen et al., (2015) found that the most common respective disease in pleural effusion and peritoneal effusion was tuberculosis and pyogenic infection. Sen et al., (2015) had one case of pericardial effusion from a patient with congestive heart failure.

The present study evaluated the utility of modified liquid-based cytology preparation for specimens classified as adenocarcinoma, malignant mesothelioma, squamous cell carcinoma, or benign mesothelial cells. We used a panel of biomarkers, including *EMA*, *Ber-EP4*, *Calretinin*, and *p63*. The 110 effusion samples (60 metastatic adenocarcinomas and 50 benign mesothelials) were prepared for immunocytochemistry using modified-LBC (Patarapadungkit et al., 2018). 

*EMA* is a protein present in carcinoma and malignant mesothelioma. *EMA* staining is mainly seen on the cell surfaces and the cytoplasm of malignant cells. Additional immunohistochemical staining should be used to differentiate mesothelial cells from carcinoma. It strongly expresses on malignant mesothelioma but not at all or weakly in reactive mesothelial cells. Several previous studies of effusion samples reported the expression of *EMA* in reactive mesothelial cells-0-14% and 75-100% of malignant mesothelioma, 100% of adenocarcinoma, and 83-100% of squamous cell carcinoma (Saad et al., 2005; Murugan et al., 2009; Ensani et al., 2011; Ikeda et al., 2011). In a recent study, all cases of adenocarcinoma showed strong membranous staining with some cytoplasmic staining. *EMA* expression was positive in 100% of adenocarcinoma cells but in none of the mesothelial cells. Similarly, Murugan et al., (2009) evaluated 39 cell blocks of adenocarcinoma effusions and 38 formalin-fixed paraffin embedded cell blocks of benign effusions that showed *EMA* was positive in 100% of adenocarcinoma cases. The *EMA* showed weak expression in cases of mesothelium (2.37%).

*Ber-EP4* is a popular adenocarcinoma marker that reacts with the two glycoproteins of the cell membrane and the cytoplasm of epithelial cells. *Ber-EP4* does not react to any significant degree with mesothelial cells or malignant mesothelioma. Several previous studies of effusion samples reported that the expression of *Ber-EP4 *in reactive mesothelial cells was 0-14% and 0-6% of malignant mesothelioma, 83-100% of adenocarcinoma cells, and 0% of squamous cell carcinoma (Robert et al., 2001; Takeshima et al., 2008; Saleh et al., 2009; Huang and Michael., 2014). In our study, *Ber-EP4* expression was positive in 91.7% of adenocarcinoma effusions vs. no expression from benign mesothelial cells. By contrast, 8.3% (5 cases) of adenocarcinoma cases were negative for *Ber-EP4* (i.e., 2 cases of breast cancer, 1 case of endometrium cancer, 1 case of lung cancer, and 1 case of unknown origin). Results from our present are consistent with previous studies. For example, Sheibani et al., (1991) studied adenocarcinomas from the breast that did not stain (32%, 8/25), and Bailey et al., (1996) evaluated 11 formalin-fixed paraffin-embedded cell blocks of adenocarcinoma effusions and 16 formalin-fixed paraffin-embedded cell blocks of benign effusions and found them 100% positive for Ber-EP4 among the adenocarcinoma cases over against none of the mesothelium cases. Ikeda et al. (2011) evaluated alcohol-fixed smears of serous effusions and found Ber-EP4 in 95% of adenocarcinoma cases vs. no expression among the mesothelium cases. 

Calretinin is a mesothelial marker which shows strong expression in benign mesothelial cells and malignant mesothelioma with a nuclear and cytoplasmic staining pattern. The respective expression of calretinin in reactive mesothelial cells and malignant mesothelioma was 93-100 and 0-20% of adenocarcinoma vs. none of the squamous cell carcinoma (Huang and Michael., 2014; Politi et al., 2005; Kim et al., 2009; Arora et al., 2011). In our study, calretinin expression was positive in 100% of mesothelial cells and in none of the adenocarcinomas. Similarly, Huang and Michael (2014) evaluated 12 formalin-fixed paraffin embedded cell blocks of adenocarcinoma effusions and 15 formalin-fixed paraffin embedded cell blocks of benign effusions, and found that calretinin expression was positive in 100% of mesothelium cases and in none of the adenocarcinoma cases. Arora et al., (2011) evaluated alcohol-fixed cytospin smears of serous effusions and found calretinin expression was positive in 90% of mesothelial cases and in none of the adenocarcinoma cases.

p63 protein is a basal epithelial cell proliferation regulator. The predominant localization of p63 protein presents in the normal basal cell and neoplastic epithelium (i.e., skin, cervix, esophagus, tonsil, urothelium, vagina, prostate, basal cells in glandular structures of breast and bronchi). The p63 protein is a nuclear marker for squamous cell carcinomas that has been utilized as the primary marker for identifying squamous cell carcinoma. Several studies of effusion samples reported the expression of p63 in reactive mesothelial cells as 0%, 0% of malignant mesothelioma, 0-14% of adenocarcinoma, and 80-100% of squamous cell carcinoma (Huang and Michael., 2014; Wu et al., 2005; Whithaus et al., 2012). In the current study, p63 expression was negative in all cases of mesothelial cells and adenocarcinoma. Similarly, Huang and Michael (2014) evaluated formalin-fixed paraffin embedded cell blocks from malignant and benign mesothelial effusions, and reported that p63 expression was negative in all cases of mesothelium but positive in 13% of adenocarcinoma cases. Wu et al., (2005) evaluated cell block samples of lung cancer patients, and reported that p63 expression was negative in all cases of adenocarcinoma. 

When combining markers (i.e., EMA, BerEp4, calretinin, and p63), it was demonstrated that 91.7% of metastatic adenocarcinomas were positive for *EMA* and *BerEp4* but negative for calretinin and *p63*. Benign mesothlial cases classified by cytomorphology were 100.0% positive for calretinin. We demonstrated that a panel marker of *EMA*, *Ber-EP4*, and calretinin can be used for differentiating between metastatic adenocarcinoma and benign mesothelial cells. The immunocytochemical panel has been studied by other researchers using different panels as well. Saleh et al., (2009) demonstrated that *BerEp4*, *MOC-31*, *HBME-1*, and calretinin were excellent biomarkers for diagnosis with 97.6% sensitivity for metastatic adenocarcinoma, and 90.7% specificity for detecting benign mesothelial cells. In addition, Murugan et al., (2009) evaluated 38 cases of benign mesothelial cells and 39 cases of metastatic adenocarcinoma, and concluded that a combination of positive for EMA and negative for calretinin or desmin had 100% sensitivity and specificity for adenocarcinoma. 

We demonstrated that a panel marker of *EMA*, *Ber-EP4*, and calretinin can be used for differentiating between metastatic adenocarcinoma and benign mesothelium cases. Serous effusion specimens were examined after applying the modified LBC, and this preparation provided effective immunocytochemical diagnosis. Immunocytochemical testing using the modified LBC smear has similar results to other studies; especially the modified LBC, which is relatively inexpensive, easy to prepare, and uses common chemical reagents. 

In conclusion, it is difficult to observe and differentiate potentially malignant or atypical cells using effusion cytology. Notwithstanding, the results of the present study support the use of effusion cytodiagnosis for routine examinations when a panel of immunocytochemistry is used to resolve problematic cases of metastatic adenocarcinoma, malignant mesothelioma, squamous cell carcinoma, or benign mesothelial cells. Herein, we demonstrated that immunocytochemical staining for *EMA*, *BerEp4*, *calretinin*, and *p63* is a useful diagnostic tool in distinguishing malignant effusions from benign effusions. 
